# Dental Convention

**Published:** 1877-09

**Authors:** 


### DENTAL CONVENTION.

The American Dental Convention, in conjunction with the Southern Dental Association and Dental Society of the State of Maryland, and District of Columbia, held a meeting at Oakland, Md. August 14-18. There were in all about seventy members present; there were also quite a number of medical gentlemen present as visitors and corresponding members. The proceedings were very interesting. The time was fully occupied in the consideration of important subjects more or less intimately connected with dental practice. Quite a number of interesting papers were read, and each freely discussed.

Without attempting to institute any comparison, or in anywise detract from the value of others, we cannot omit to mention two papers, the one on the "Power and Uses of the Spectroscope," by S. Waterman, M. D., of New York, and the other by J. J. Caldwell, M. D., of Baltimore, on the "The Involuntary Action of the Nervous System."

The former, showing the wonderful revelations of the specroscope and the wide reach and range of its use. It is one of the most valuable aids in medical diagnosis, reaching into fields that have hitherto been in total obscurity, and making revelations that no other instrumentality has accomplished.

Dr. Caldwells paper will be found in the present number of the REGISTER, it will repay a careful perusal.

The Convention and the Southern Association each appointed a committee to confer with the committee from the American Dental Association to arrange for a consolidated organization, thus bringing into one body that which has for so long been in a fragmentary condition. These three bodies meet simultaneously at Niagara Falls in August next, when there will probably be the largest gathering of the dental profession ever held in this country.

